# Supplementary material for: Internet Access and Use by Patients with Gynecologic Malignancies: A Cross-Sectional Study
Source: Cancers (Basel). 2024 Apr 26;16(9):1677. doi: 10.3390/cancers16091677 (PMC11083592; doi:10.3390/cancers16091677)
Supplement: Supplementary file 1 [file cancers-16-01677-s001.zip › cancers-2882873-supplementary.pdf]

Supplementary Table S1

			Age				Education			Cancer entity			
		**All**	**<49 years**	**50-59 years**	**60-69 years**	**>70 years**	**Low**	**Middle**	**High**	**Ovarian Cancer**	**Cervical Cancer**	**Vulvar cancer**	**Endometrial Cancer**
Computer property		133/146 (91.1%)	37/38 (97.4%)	42/45 (93.3%)	38/42 (90.5%)	16/21 (76.2%)	15/20 (75%)	41/46 (89.1%)	76/79 (96.2%)	41/46 (89.1%)	37/39 (94.9%)	30/33 (90.9%	24/28 (89.3%
Computer experience	no	11/147 (7.5%)	0/38 0%	2/44 (4.5%)	5/43 (11.6%)	4/22 (18.2%)	6/21 (28.6%)	4/46 (8.7%)	1/79 (1.3%)	4/48 (8.3%)	2/39 (5.1%)	3/33 (9.1%)	2/27 (7.4%)
	low	23/147 (15.6%)	0/38 0%	7/44 (15.9%)	8/43 (18.6%)	8/22 (36.4%)	6/21 (28.6%)	9/46 (19.6%)	7/79 (8.9%)	11/48 (22.9%)	3/39 (7.7%)	2/33 (6.1%)	7/27 25.9%)
	good	79/147 (53.7%)	20/38 (52.6%)	27/44 (61.4%)	24/43 (55.8%)	8/22 (36.4%9	9/21 (42.9%)	25/46 (54.3%)	45/79 (57%)	26/48 (54.2%)	18/39 (46.2%)	23/33 (69.7%)	12/27 44.4%)
	very good	34/147 (23.1%)	18/38 (47.4%)	8/44 (18.2%)	6/43 (14%)	2/22 (9.1%)	0/21 0%	8/46 (17.4%)	26/79 (32.9%)	7/48 (14.6%)	16/39 (41%)	5/33 (15.2%)	6/27 22.2%)
Computer use	Work	8/132 (6.1%)	5/37 (13.5%)	2/41 (4.9%)	1/38 (2.6%)	0/16 0%	0/16 0%	2/39 (5.1%)	6/76 (7.9%)	1/41 (2.4%)	4/37 (10.8%)	1/28 (3.6%)	2/26 (7.7%)
	Home	56/132 (42.4%)	5/37 (13.5%)	16/41 (39%)	23/38 (60.5%)	12/16 (75%)	14/16 (87.5%)	20/39 (51.3%)	21/76 (27.6%)	21/41 (51.2%)	11/37 (29.7%)	12/28 (42.9%)	12/26 (46.2%)
	Work + Home	68/132 (51.5%)	27/37 (73%)	23/41 (56.1%)	14/38 (36.8%)	4/16 (25%)	2/16 (12.5%)	17/39 (43.6%)	49/76 (64.5%)	19/41 (46.3%)	22/37 (59.5%)	15/28 (53.6%)	12/26 (46.2%)
Phone without internet		94/133 70.7%	14/34 41.2%	26/38 68.4%	32/39 82.1%	22/22 100%	16/18 88.9%	32/44 72.7%	44/69 63.8%	34/44 77.3%	15/31 48.4%	24/31 77.4%	21/27 77.8%
Phone with Internet/ Smartphone		114/133 85.7%	34/39 87.2%	33/38 86.8%	33/39 84.6%	14/22 63.6%	13/18 72.2%	37/44 84.1%	63/69 91.3%	38/44 86.4%	28/31 90.3%	25/31 80.6%	23/27 85.2%
Tablet		43/132 32.6%	15/34 44.1%	11/37 29.7%	10/39 25.6%	7/22 31.8%	0/18 0%	14/44 31.8%	29/68 42.6%	12/43 27.9%	15/31 48.4%	8/31 25.8%	8/27 29.6
Internet access		138/144 (95.8%)	38/38 (100%)	41/42 (97.6%)	40/43 (93%)	19/21 (90.5%)	18/20 (90%)	42/44 (95.5%)	77/79 (97.5%)	44/46 (95.7%)	38/38 (100%)	31/33 (93.9%)	25/27 (92.6%)
Internet use	By themselves	133/145 (91.7%)	38/38 (100%)	42/44 (95.5%)	36/42 (85.7%)	17/21 (81%)	16/21 (76.2%)	40/44 (90.9%)	76/79 (96.2%)	43/48 (89.6%)	36/38 (94.7%)	29/32 (90.6%)	25/27 (92.6%)
	Indirectly via friends/ family	3/145 (2.1%)	0/38 0%	0/44 0%	2/42 (4.8%9	1/21 (4.8%)	1/21 (4.8%)	1/44 (2.3%)	1/79 (1.3%)	2/48 (4.2%)	1/38 (2.6%)	0/32 0%	0/27 0%
	no	9/145 (6.2%)	0/38 0%	2/44 (4.5%)	4/42 (9.5%)	3/21 (14.3%)	4/21 (19%)	3/44 (6.8%)	2/79 (2.5%)	3/48 (6.3%)	1/38 (2.6%)	3/32 (9.4%)	2/27 (7.4%)
Frequency of internet use	Daily	107/139 (77%)	37/38 (97.4%)	35/43 (81.4%)	25/40 (62.5%)	10/18 (55.6%)	8/18 (44.4%)	28/42 (66.7%)	71/78 (91%)	31/46 (67.4%)	33/37 (89.2%)	23(30 (76.7%)	20/26 (76.9%)
	Several times/ week	22/139 (15.8%)	1/38 (2.6%)	8/43 (18.6%)	8/40 (20%)	5/18 (27.8%)	5/18 (27.8%)	10/42 (23.8%)	6/78 (7.7%)	10/46 (21.7%)	3/37 (8.1%)	5/30 (16.7%)	4/26 (15.4%)
	Several times/ month	5/139 (3.6%)	0/38 0%	0/43 0%	3/40 (7.5%)	2/18 (11.1%)	1/18 (5.6%)	3/42 (7.1%)	1/78 (1.3%)	3/46 (6.5%)	1/37 (2.7%)	1/30 (3.3%)	1/26 (3.8%)
	Less than 1x/month	5/139 (3.6%)	0/38 0%	0/43 0%	4/40 (10%)	1/18 (5.6%)	4/18 (22.2%)	1/42 (2.4%)	0/78 0%	2/46 (4.3%)	0/37 0%	1/30 (3.3%)	1/26 (3.8%)
